# Brain fluid physiology in ischaemic stroke; more than just oedema

**DOI:** 10.1186/s12987-025-00671-8

**Published:** 2025-06-18

**Authors:** Kirsten G. Coupland, Merce Fuentes Amell, Neil J. Spratt

**Affiliations:** 1 School of Biomedical Sciences and Pharmacy, The University of Newcastle Australia, Newcastle, Australia; 2 Heart and Stroke Program, Hunter Medical Research Institute, Newcastle, Australia; 3 Department of Neurology, John Hunter Hospital, Newcastle, Australia

**Keywords:** Cerebrospinal fluid, Stroke, Stroke pathophysiology, Cerebral oedema, Intracranial pressure, Glymphatics, Cerebrospinal fluid efflux

## Abstract

**Background:**

Cerebrospinal fluid and interstitial fluid dynamics are critical for maintaining homeostasis in the central nervous system. These fluids facilitate waste clearance, micronutrient distribution, and provide a tightly regulated ionic environment. Ischaemic stroke, a leading cause of morbidity and mortality, disrupts this delicate system, compounding the physiological challenges posed by the condition. Despite recent advances in our understanding of the importance of cerebrospinal fluid (CSF) and interstitial fluid (ISF) movement and exchange, the role of this system in stroke pathophysiology remains underexplored.

**Main body:**

Emerging evidence indicates that ischaemic stroke acutely alters CSF and ISF movement and exchange, with effects observed at both local and brain-wide levels. In the hyper-acute phase, there is an influx of CSF into perivascular spaces, potentially contributing to early cell swelling. Over time, impaired clearance mechanisms exacerbate ionic and vasogenic oedema, elevating intracranial pressure and further compromising perfusion in the ischaemic penumbra. Mechanistic studies suggest that disruptions in arterial pulsatility, extracellular space microstructure, and aquaporin 4 localisation may underlie these changes. Experimental models have revealed decreased CSF and ISF exchange, movement and outflow in the hours to days following stroke, with implications for waste clearance and secondary injury processes. The interplay between these dynamics and cortical spreading depolarisations, stroke severity, and cerebrovascular physiology adds complexity to understanding the condition’s progression.

**Conclusion:**

The disruption of CSF and ISF movement and exchange may represent a significant, yet underappreciated contributor to post-stroke pathology. Addressing these alterations could offer novel therapeutic avenues to mitigate secondary damage, improve central nervous system (CNS) homeostasis, and enhance recovery outcomes. Future research must focus on elucidating the precise mechanisms of CSF and ISF movement and exchange disturbance and exploring targeted interventions to restore normal fluid dynamics in the CNS post-stroke.

## CSF and ISF movement and exchange

CSF is derived from blood and is predominantly produced by the four choroid plexuses; one located in each of the cerebral ventricles. The choroid plexuses are highly vascularised organs that produce CSF [1, 2] purportedly via a series of ion co-transport channels that transport water alongside solutes [3]. A portion of CSF volume may be derived from the ISF that occupies the extracellular space (ECS). The proportion of CSF produced by the choroid plexuses versus ISF from other sources is difficult to quantify. Reports indicate a wide range of CSF to ISF ratios; 40–70% has been reported to be derived from the choroid plexuses, and 30–60% is derived from the ISF, a figure which may vary between species and is an area of ongoing debate [1, 4–6]. The weight of evidence indicates that CSF is primarily produced by the choroid plexuses with some additional brain transcapillary water exchange and metabolic processes contributing to CSF production [7]. From a practical point of view, there is interchange between these two fluid compartments and their composition is very similar [8].

**Fig. 1:**
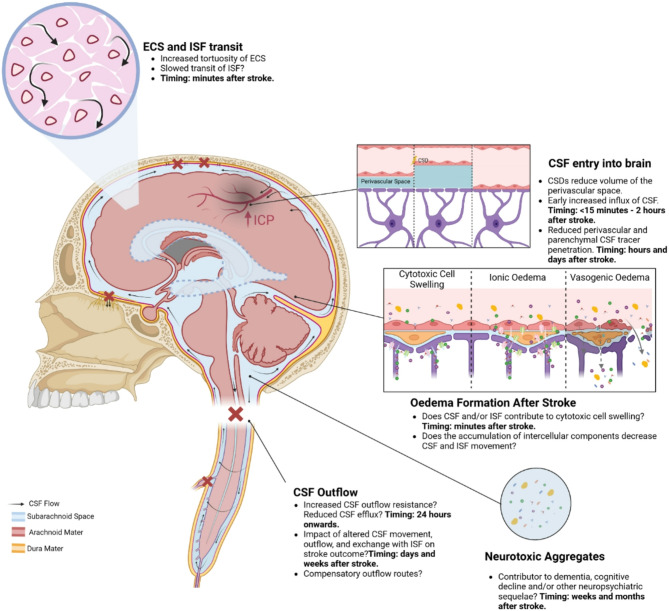
An overview of our current, broad understanding of the production and net movement of CSF. CSF is predominantly produced in the choroid plexuses of the four ventricles and from there migrates into either the periventricular space or the subarachnoid space of the brain and spinal cord via the aqueduct of Sylvius and the foramens of Luschka and Magendie. CSF then permeates the brain via Virchow-Robin spaces around penetrating arteries and by crossing the pia mater to enter the parenchyma where it mixes with ISF. Evidence indicates that outflow of the mixed CSF and ISF appears to occur via perivenous spaces (glymphatic hypothesis), but there is some evidence that it may occur through the arterial basement membrane (IPAD hypothesis). From the subarachnoid space CSF exits the cranial compartment via several routes including around cranial and spinal nerves and the meningeal lymphatics, particularly those at the cribriform plate (reviewed in 18) where it then returns to the peripheral circulation. Created in BioRender. Spratt, N. (2025) https://BioRender.com/y18u546

CSF and ISF are involved in waste removal from the central nervous system (CNS) and provide a tightly regulated fluid for intercellular communication and CNS homeostasis. In vivo imaging techniques to non-invasively assess the movement and exchange of CSF and ISF continue to improve but the exact movement patterns of these fluids remain incompletely understood and net movement cannot be definitively described. Broadly, CSF arising from the choroid plexuses moves through the ventricular system towards the base of the brain or permeates the periventricular parenchyma via gap junctions in the ependyma to mix with ISF [9] (Fig. 1). CSF then moves into the subarachnoid space of the brain and spinal cord. CSF permeates the brain via Virchow-Robin spaces surrounding penetrating arteries and arterioles, which are in continuity with the subarachnoid space. CSF moves into the ECS of the parenchyma, where it mixes with the ISF, in two ways: from the perivascular space, and from the subarachnoid space by crossing the pia mater and glia limitans. Within the parenchyma, ISF movement is dictated by either diffusion, convection, or a combination of the two. The weight of evidence indicates that diffusive forces dictate ISF tracer distribution in the parenchyma, but there is some evidence that convective forces may also contribute (see [10] for further detail). CSF and ISF flow is also subject to back-and-forth oscillations due to arterial and respiratory rhythms [11–13]. How ISF moves out of the parenchyma remains an area of ongoing debate. While there is little debate that periarteriolar and/or Virchow-Robin spaces are a route of CSF tracer influx, there is much more controversy about the primary outflow route. There are two dominant theories: the glymphatic system [14], and intramural periarterial drainage (IPAD) [15]. The purported glymphatic pathway involves aquaporin 4 (AQP4)- with convective flux-dependent uptake of CSF at the capillary bed and CSF and/or ISF efflux via peri-venous outflow routes [14, 16]. IPAD describes CSF and/or ISF efflux via the basement membrane of arterial smooth muscle cells in arteries [15]. In addition to these two models, there is evidence of CSF and/or ISF movement via convection along fibre tracts in white matter [4]. It remains to be definitively established whether one, all three, or an entirely different model explains CSF and/or ISF efflux from the deep brain structures. In support of perivenous outflow, recently published work indicates that structures around the outside of bridging veins termed ‘arachnoid cuff exits’ allow CSF solutes to drain from the subarachnoid space into the epidural spaces adjacent to dural venous sinuses [17]. In addition, a time-course analysis of intracisternally injected tracer distribution identified that the tracer is found in perivenous spaces at 120 minutes after tracer injection but not at earlier timepoints, indicating the tracer likely migrates to the perivenous space from the subarachnoid space [16]. Once in the subarachnoid space, the mixed CSF and ISF exits the craniospinal compartment via several routes including around cranial and spinal nerves and the meningeal lymphatics, particularly those at the cribriform plate, though again our understanding of the relative contribution of each out these outflow routes is incomplete particularly given that our current understanding is mainly drawn from animal studies (see [18] for further discussion). Once the CSF has exited the cranio-spinal cavity it returns to the peripheral circulation [14, 19].

In examining CSF and ISF dynamics in experimental models, there are several important methodological details to consider:

- Tracer size: tracers of differing molecular weights exhibit different distribution patterns in the perivascular space and parenchyma. Small solutes of ≤ 10 kDa readily penetrate the parenchyma from the ventricles [9], perivascular space, and the cortical surface in contact with the subarachnoid space [14], while solutes of a moderate size (~ 20–60 kDa) enter the parenchyma by the same routes but at a slower rate [14, 19], hinting that diffusive forces dictate solute movement in the intercellular space [20]. Larger solutes (> 1000 kDa) appear to be restricted to the subarachnoid and perivascular spaces with limited movement into the parenchyma [14]. Solute size does not influence the rate of solute transit in the perivascular space indicating that bulk flow is the dominant transport force [20].
- Anaesthesia choice: examining the movement of CSF and ISF often necessitates the use of general anaesthesia. CSF and ISF movement and exchange are differentially impacted by different methods of general anaesthesia. The most commonly used inhalation anaesthetic in experimental studies, isoflurane, appears to reduce perivascular influx of CSF tracer in vivo while ketamine/xylazine recapitulates CSF and ISF movement similar to those seen in sleep [21, 22]. Supplementation of isoflurane anaesthesia with the α2-adrenergic receptor agonist dexmedetomidine results in movement of these fluids being similar to those observed in sleeping animals [23, 24]. It should be noted that the reported differences in CSF and ISF movement during wake/sleep and anaesthesia could be due to altered CSF outflow which in turn affects the movement of CSF and ISF in the brain [25].
- Injection location: there are three injection sites commonly used in studies examining CSF and ISF tracer movement: the lateral ventricles, the cisterna magna, and the parenchyma. Each tracer injection site allows for interrogation of fluid movement at different stages of tracer transit. Injections of tracer into the parenchyma are used to understand routes of solute efflux from the ECS of brain, while those into the cisterna magna and the ventricles are preferred when CSF tracer influx into the parenchyma and/or CSF efflux pathways out of the craniospinal compartment are being investigated. Interestingly, some reports indicate that the movement of tracers injected into the ventricles differ from those injected into the cisterna magna. Iliff et al., [14] reported that CSF tracer load in the parenchyma was lower when tracer was injected into the lateral ventricles as opposed to the cisterna magna 30 min after injection. In addition, Ma et al., [25] reported that tracer injected intraventricularly was enriched in the sacral spine at a faster rate than in those that received intracisternal injections, indicating that tracers injected intraventricularly likely move preferentially to the central canal of the spine. Earlier reports however demonstrate tracer penetration into the perivascular space within 10 min of intracerebroventricular injection [26]. It is possible that the difference in CSF tracer transit routes is attributable to different rates of injection (see next dot point). A side-by-side temporal comparison of the two tracer injection methods using [C^14^]inulin reported that the only major difference in tracer distribution was the lack of tracer load in the ventricles in brain tissue from animals that received intracisternal injections [9].
- Injection rate and osmolarity: injection of tracers into the CSF or ISF alters the delicate dynamics of CSF and ISF transit and exchange. Greater injection rates into the cisterna magna have been demonstrated to decrease the time taken for tracers to penetrate the perivascular space and enter the parenchyma [27]. Recent estimates of CSF production rates in mice place production rates at ~ 0.32 µl/min, but it should be noted that the method used for this estimate did not include CSF production by the fourth ventricle [28]. For mice, infusion rates at or below 0.7 µl/min have been demonstrated not to affect intracranial pressure [28]. It is also vital to consider the osmolality of the solvent used for tracer infusion. The local osmotic environment is important in the production of CSF and use of osmotic solutions can cause disruption to this system [29].
- In vivo vs. ex vivo: CSF tracer signal in the perivascular space of penetrating vessels has been demonstrated to increase after death [30]. Furthermore, the pial perivascular space has been demonstrated to collapse upon paraformaldehyde fixation [31]. Both these phenomena have the potential to confound tracer location and concentration in ex vivo studies. However, ex-vivo studies have the advantage of whole brain imaging, whereas in vivo studies tend to be restricted to the cortex at the vertex or a small subcortical region via 2-photon imaging. Transit of CSF tracers appears to be much lower to the parenchyma at the vertex than elsewhere in the brain, particularly in comparison to the basal cisterns and therefore may not be an accurate representation of CSF and ISF movement [9, 32, 33]. Attempts to monitor CSF movement in vivo using MRI have had some success. Some studies examined CSF movement using an intrathecal tracer [34–39], while others developed non-contrast imaging modalities [40]. The imaging modality to gain the most traction to date is diffusion tensor image analysis along the perivascular space (DTI-ALPS) which appeared to identify a reduction in CSF movement in the ipsilateral hemisphere of stroke patients when compared to the contralateral hemisphere [41]. It should, however, be noted that follow-up studies that deployed DTI-ALPS have indicated that this technique may not be measuring glymphatic function [42–45]. It should also be noted that, while these imaging modalities are useful to understand alterations to CSF movement, they have not been able to provide insights into net ISF movement.

### CSF and ISF movement, production, and exchange in disease: what we already know

Maintenance of CSF and ISF movement, production, and exchange is essential for central nervous system homeostasis. Disruption of this system is associated with several pathologies. Obstructive and non-obstructive hydrocephalus are conditions that result in accumulation of CSF in the cranial compartment. Obstructive, or non-communicating, hydrocephalus occurs due to stenosis of the cerebral aqueduct or in the presence of a volume-occupying lesion that restricts efflux of CSF from the ventricles (e.g., a tectal plate glioma). Non-obstructive, or communicating, hydrocephalus involves altered CSF movement once it exits the ventricles [46]. This condition arises due to altered outflow of CSF from the cranial space or, in rare instances, due to overproduction of CSF. Communicating hydrocephalus can occur due to congenital factors or as a complication of subarachnoid haemorrhage, or infectious or carcinomatous meningitis. In the latter two, the common factor thought to affect CSF outflow is inflammation and scarring of the meninges which could impede CSF outflow at efflux pathways [47]. Communicating hydrocephalus is not always associated with raised intracranial pressure, in which case it is referred to as normal pressure hydrocephalus [48, 49]. As implied by its name, normal pressure hydrocephalus is not associated with increased intracranial pressure, nor with any observable obstruction to CSF movement. There are two forms of normal pressure hydrocephalus: secondary normal pressure hydrocephalus, which tends to be observed in patients with prior traumatic brain injury, infection, haemorrhage, or radiation exposure, and idiopathic normal pressure hydrocephalus the pathology of which remains unclear but its incidence increases with age [50, 51]. Patients with normal pressure hydrocephalus may improve with insertion of a shunt to drain excess CSF [52], however identification of those most likely to benefit from shunt insertion remains a topic of great research interest [53].

Idiopathic intracranial hypertension is a condition also thought to be associated with CSF flow disturbance. There are no structural lesions, although clinical presentation is otherwise quite similar to that of patients with cerebral venous thrombosis, and at least a subset of those with idiopathic intracranial hypertension have evidence of dynamic venous sinus stenosis that improves after pressures are lowered by large volume lumbar puncture for CSF drainage [54]. The epidemiology is remarkable for the very strong association with overweight women of childbearing age and recently published research indicates that obesity-induced androgen dysregulation may contribute to this condition [55]. However, the exact pathogenesis is yet to be elucidated.

These conditions highlight the importance of normal CSF and ISF movement, production, and efflux in the maintenance of central nervous system homeostasis. For most of the past few decades, the above conditions have made up the majority of clinically recognised conditions involving disruption to CSF movement, production, and efflux. Renewed interest in the field of CSF and ISF movement, production, and efflux in the past decade has seen interesting studies indicate that this system may play a role in the primary and secondary pathology of other, more common conditions, including Alzheimer’s disease and ischaemic stroke. This review will assess the evidence for disrupted CSF dynamics after ischaemic stroke and how it may impact stroke sequelae.

### Ischaemic stroke

CSF and ISF movement, production, and efflux after ischaemic stroke has received only limited study, to date. While there are published studies that examine CSF and ISF dynamics after ischaemic stroke from which rational hypotheses can be extrapolated, they remain piecemeal. In the below sections we will outline what we know, what is likely, and where the open questions for future investigation lay.

The handful of studies that have examined the impact of ischaemic stroke on CSF circulation mostly report a transient decrease in CSF tracer signal intensity in the perivascular space and/or the parenchyma of the ipsilateral hemisphere after stroke indicating that ischaemic stroke disrupts normal CSF movement, and CSF to ISF exchange [32, 56–58]. It should be noted that Mestre et al., [59] report a transient increase in perivascular CSF tracer after stroke, which is discussed at length in “*Timing and location of disrupted CSF movement and CSF an ISF exchange after stroke”*. Differences in the findings reported by these studies include location, duration, and timing of altered CSF movement (Table 1). In this section of the review, we will provide a broad overview of what has been reported in the literature which will be expanded upon in greater detail in subsequent sections.

**Table 1:**
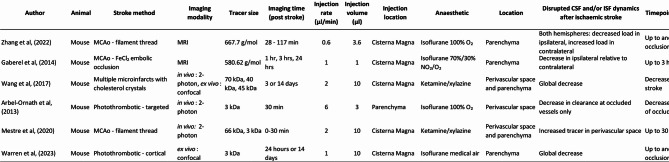
Summary of published studies examining CSF and/or ISF dynamics after ischaemic stroke

#### Timing and location of disrupted CSF movement and CSF to ISF exchange after stroke

Studies that examine the location of disrupted CSF movement and CSF to ISF exchange in relation to stroke injury differ in their findings, with some indicating a disruption proximal to the injury, while others report a brain-wide perturbation. There are also temporal differences in CSF movement after stroke, with a rapid influx of CSF occurring in the minutes following stroke [59, 60], and a decrease in CSF tracer influx into the perivascular spaces and/or parenchyma in the hours to days following stroke [32, 56, 61]. Mestre et al., [59] characterised a sudden influx of cisternally administered tracer into the perivascular space in the minutes following vessel occlusion. The authors posit that this is due to vasoconstriction, which appeared to be temporally correlated with stroke-derived cortical spreading depolarisations (CSDs), resulting in concurrent expansion of available perivascular space (note: the impact of CSDs on CSF movement is further explored in the section entitled “*Evidence that cortical spreading depolarisation disrupts CSF and ISF movement*”). By 15 min after middle cerebral artery occlusion (MCAo) the volume of the perivascular space appears to be comparable to that of sham animals, and between 15 and 30 min there is a decrease in perivascular volume such that it is smaller than in sham animals. Whether the CSF tracer migrates into the parenchyma from the perivascular space to mix with ISF as a result of the decreased perivascular space at > 15 min after stroke is unclear. In a separate study, increased uptake of intracisternally injected tracer (Gd-BOPTA) into the parenchyma relative to sham was reported between 28 and 117 min (the experimental endpoint) after permanent MCAo [60] indicating that increased CSF influx into the ipsilateral hemisphere persists until at least two hours after stroke. One study noted decreased clearance of intraparenchymal ISF tracer from the area around an occluded vessel in the 30 min immediately following vessel occlusion [58]. This indicates that there may be a decreased efflux of ISF from the parenchyma alongside an increase in CSF influx into the perivascular space. Reduced CSF influx into the perivascular space and/or parenchyma has been reported to occur at later timepoint of between three to 24 h after stroke [32, 56], with one study reporting that this decrease in influx persisted up to three days after stroke [57]. In support of this, one study reported reduced CSF transit in the perivascular space of the ipsilateral hemisphere 24 h after thrombectomy in stroke patients [62]. It is feasible that maximal dilation of vessels in hypoperfused tissue may mean that available perivascular space for CSF influx is reduced. If this is true, then chronic dilation of cerebral arteries may lead to a persistent reduction in perivascular space that restricts movement of CSF into the perivascular space, potentially explaining the observed decrease in CSF tracer influx at later timepoints.

As indicated earlier, most studies have noted a decrease in CSF and/or ISF tracer load in the parenchyma and/or perivascular space after stroke, however, the span of affected regions in relation to injury site differs with some indicating a hyper-localised effect close to the occluded vessel, while others report a brain-wide decrease in CSF and ISF movement. Some reports indicate that disruption to CSF and ISF movement and exchange are localised to either the ipsilateral hemisphere [56, 60] or the perivascular space surrounding the occluded vessel [58]. Other studies have reported reduced CSF or ISF tracer load in both hemispheres, indicating that disruption may occur in areas distal to the injury. It is worth noting that the multiple microinfarct model used by Wang et al., [57, 61] has previously been demonstrated to result in microinfarcts in the contralateral hemisphere which could partially explain the global reduction to tracer distribution in the parenchyma. Another study that reported a global decrease in tracer distribution used the photothrombotic model of stroke [32]. Given that this model results in a focal cortical lesion it is unlikely to affect cerebral haemodynamics in the contralateral hemisphere. It is possible that the embolic stroke model used by Gaberel et al., [56] also resulted in a global decrease in CSF and ISF tracer distribution; indeed, images included in the publication appear to suggest that this might be the case. However, as their analysis used the contralateral hemisphere as the control ‘uninjured’ tissue, it is not possible to determine whether there is a decrease in tracer distribution in the contralateral hemisphere relative to sham tissue. The mechanisms underlying whole brain disruption of CSF and ISF movement and exchange, and whether this is reflective of what is seen in patients, warrants further investigation. Regardless of which regions of the brain are affected, it is reassuring that, despite the use of different methods, most studies report an influx of CSF tracer into the perivascular space in the minutes following stroke, while, from three hours onwards there is a reduction in CSF tracer influx into these spaces in either the ipsilateral hemisphere or the whole brain after stroke. The reported differences in the duration and location of disrupted CSF and ISF dynamics after stroke need to be investigated via systematic experiments using different stroke models such that model-specific perturbations to CSF and ISF dynamics can be excluded from the current working model.

#### Duration of disruption to CSF and ISF movement after stroke

How long after stroke CSF and ISF movement remains disrupted is not definitively established but all studies that provide follow-up analyses report that CSF and ISF movement and exchange are restored at later timepoints. CSF and ISF movement has been reported to be restored as soon as 24 h after stroke [56], with others indicating that the system remains perturbed until at least 24 h or three days after stroke [32, 57]. Two studies have reported that CSF tracer influx into the parenchyma is comparable to sham operated animals by two weeks after injury. These differences in findings likely arise from methodological restrictions in continuous imaging of CSF movement which confines analyses to specific timepoints. Gaberel et al. (2014) found that CSF movement was restored 24 h after stroke coinciding with vessel patency as determined by MRI [56]. Other studies reported more persistent disruptions to CSF and ISF tracer movement which extended out to 24 h [32] and three days [57] after stroke. These studies did not examine whether vessel patency was a factor in the restoration of CSF movement. Given that the work by Gaberel et al., [56] is the only report thus far that has examined CSF tracer movement after vessel recanalization, it may be that reperfusion restores CSF movement. Certainly, the two studies that report an extended timeframe of disruption to CSF movement after stroke used permanent vessel occlusion models [32, 57]. In these permanent occlusion models, both Warren et al., [32] and Wang et al., [57] reported restoration of CSF tracer distribution by two weeks after stroke, indicating that, regardless of vessel patency, post stroke alterations to CSF movement resolve by two weeks after stroke. As with our current understanding of the timing and location of disruptions to CSF and ISF movement after stroke, understanding how long after stroke disruptions to this system persist should deploy different models, modes of general anaesthesia, and tracers.

### The contributions of CSF and ISF to post stroke oedema

The Monroe-Kellie doctrine outlines that there are three volume-occupying components in the cranial space: blood, brain tissue, and CSF and ISF [63, 64]. As the cranial space is strictly limited by the skull, a significant increase in any one of these must be compensated by a decrease in volume of at least one of the others to prevent elevated ICP. Oedema, of which there are two putative forms; vasogenic and ionic, with some schools of thought highlighting cytotoxic cell swelling as a potential third form, has classically been considered the main driver of elevated ICP after stroke, however, not all forms contribute to ICP elevation after stroke [65]. Cytotoxic cell swelling and ionic oedema occur while the blood brain barrier is intact, while vasogenic oedema is principally derived from the vascular compartment after disruption of the blood brain barrier. Ionic and vasogenic oedema both lead to tissue swelling and mass effect. For a recent, detailed review of the physiology of oedema formation after ischaemic stroke readers are directed to Hladky and Barrand. (2024) [66].

#### Cell swelling

At present there is ongoing debate as to the proper classification of different forms of oedema that occur after ischaemic stroke (see [67] and footnote 1 of [66]). To allow for detailed assessment of the sequential steps in the generation of cerebral oedema, we have opted to discuss cell swelling as a process that precedes and coincides with the movement of salts and water across the BBB to form cytotoxic/ionic oedema in line with Simard and colleagues [65, 67–69]. Early cell swelling occurs as a result of Na^+^, accompanied by Cl^−^ and water from the ISF, moving from the ECS to the intracellular space in the minutes following occlusion. As a result, there is no net change in volume within the cranial compartment and cell swelling does not alter ICP in the minutes immediately following stroke onset [65]. The concomitant transit of water and osmolytes from the vascular compartment to the CNS, which further contributes to cell swelling and can alter ICP, is discussed at length in the next section entitled “Cytotoxic/Ionic Oedema”. There appear to be several mechanisms that drive this intracellular influx of osmolytes and water, all involving movement of CSF and ISF into the intracellular compartment. Cytotoxic cell swelling occurs due to failure of ATP-dependent ion pumps such as Na^+^/K^+^ ATPase [70] and ion transport channels that function independently of ATP, such as the Na^+^/K^+^/Cl^−^ co-transporter (NKCC1) [71–74]. The failure of these pumps leads to the intracellular accumulation of osmolytes, and subsequent uptake of Cl^−^ and water to maintain electroneutrality. Failure of ATP production after stroke leads to a cascade of events that may impact on CSF and ISF movement and exchange, including cell swelling, which likely contributes to the increased tortuosity of the ECS discussed in “Impacts of ischaemic stroke on ISF movement and exchange with CSF in the parenchyma”. There is also evidence that excitatory amino acid transporters that take up extracellular glutamate, released as a result of spreading depolarisation or neuronal lysis after stroke, form complexes with aquaporin 4 (AQP4), the water transport channel constitutively expressed by astrocytes, which facilitate co-transport of water alongside glutamate, leading to astrocytic swelling [75–77]. A fourth contributor to cytotoxic cell swelling, that is also involved in the formation of cytotoxic/ionic oedema, is the injury-induced formation of SUR1-TRPM4-AQP4, a heteromultimeric water/ion channel that opens in response to low levels of ATP allowing influx of K^+^, Na^+^ and water into endothelial cells, neuronal cells, and astrocytes [78–82]. The re-localisation of water and solutes from the ECS to the intracellular space creates an osmotic gradient that draws water and ions from the vascular compartment into the extracellular space, contributing to the generation of cytotoxic/ionic oedema (see section entitled “Cytotoxic/ionic oedema”).

Following Simard and colleagues model of early cell swelling [67], it is most likely that CSF and ISF are the primary source of water and osmolytes, with water and ions from the vascular compartment as another potential source. Recent work by Mestre et al., [59] which demonstrated influx of CSF tracer into the perivascular space within minutes of occlusion in a mouse model of stroke seems to support this hypothesis. The authors noted an influx of CSF into the Virchow-Robin spaces approximately five minutes after occlusion, that coincided with spreading depolarisation (discussed in further detail below, in “Evidence that cortical spreading depolarisation disrupts CSF and ISF movement”). Quantitation of water weight between 15 and 60 min after stroke found elevated water content in the ipsilateral hemisphere, indicating that CSF had likely been taken up by cells, although it should be noted that cellular uptake of CSF tracer was not directly demonstrated. To determine whether the water and osmolytes that contributed to oedema formation in this model were derived from the vascular compartment or CSF and/or ISF, radiolabelled ^22^Na^+^ and ^3^H-mannitol were injected intravenously prior to stroke induction and the resultant abundance of the tracers in the ipsilateral and contralateral hemispheres measured. While radiolabelled ^22^Na^+^ was present in the brain 15 min after MCAo, it was comparable between hemispheres and the increase in weight of the ipsilateral hemisphere is not fully accounted for by the increase in sodium uptake from the blood [59]. When the tracers were injected intracisternally, both tracers accumulated in the ipsilateral hemisphere, indicating that CSF and/or ISF is a key contributor to early cell swelling in the first 15 min after stroke [59]. At later timepoints, evidence indicates that the water and ions that drive the formation of cytotoxic/ionic oedema is most likely derived from vascular sources.

#### Cytotoxic/ionic oedema

Water and osmolytes that contribute to cytotoxic/ionic oedema are thought to be derived from the capillaries without concurrent movement of vascular proteins as the capillary endothelial layer is permeable to water in a bidirectional manner in the healthy brain [65, 83]. As water and osmolytes move from the extracellular to the intracellular space during early cell swelling, it is feasible to suggest that disproportionate water movement results in the establishment of an osmotic gradient relative to plasma. Such a gradient could draw water across the luminal wall into the parenchyma [84]. Other teams have suggested that net flux of ions from plasma to the ISF is sufficient to maintain the iso-osmotic state and that instead the osmotic gradient is established due to the accumulation of metabolites derived from cellular respiration and/or the production of lactic acid due to anaerobic metabolism in ischaemic tissue [85, 86]. While the size of the osmotic gradient established between the CSF and blood after stroke has not been extensively examined, one study involving experimental stroke in rats reported that brain osmolality increased from 311 ± 2 mOsm/kg to 329 ± 2 mOsm/kg within 6 h post-occlusion, while serum osmolality remained relatively stable. This resulted in a peak osmotic gradient of approximately 26 mOsm/kg between the blood and brain. By 12 h after occlusion, brain osmolality decreased to 310 ± 2 mOsm/kg and maintained that level thereafter [87].

An alternative hypothesis is that CSF and/or ISF may be a contributor to cytotoxic/ionic oedema. For this to be the case there would have to be additional CSF or ISF volume in the cranial compartment. This could occur through increased CSF or ISF production or reduced outflow. While there is no evidence of increased CSF production after stroke (in fact, the opposite has been demonstrated [59, 88]), studies have demonstrated increased CSF outflow resistance after stroke leading to reduced outflow after experimental stroke [88, 89]. Other studies have demonstrated reduced outflow of CSF tracers to the nasal mucosa and the deep cervical lymph nodes from the cranial compartment after stroke, indicating that outflow of CSF is reduced at these locations [32, 90, 91]. This could lead to a build-up of CSF and/or ISF in the cranial compartment and potentially provide a source of additional ions and fluids for the formation of cytotoxic/ionic oedema. Recently published findings indicate that potentiating CSF drainage through the meningeal lymphatics reduces oedema volume after experimental traumatic brain injury in rodents, indicating that drainage of fluids from the craniospinal compartment reduces the availability of fluids necessary for oedema formation [92]. The contributions of either or both of these pathways to cytotoxic/ionic oedema remains to be definitively established.

#### Vasogenic oedema

The contribution of CSF to vasogenic oedema is minimal, with the majority of water coming from the vascular compartment due to breakdown of the blood brain barrier. Instead, there is evidence that vasogenic oedema impacts on CSF and ISF movement due to mass effect and elevated ICP, which is discussed in detail in the next section. The global decrease in CSF and ISF tracer distribution at three days after stroke noted by Wang et al., [57] could be due to the presence of space-occupying vasogenic oedema which peaks 48–72 h after stroke [93], although, given that this study used a model of microinfarcts, one would not anticipate large oedema volumes. In the absence of quantitation of oedema in this study, it is not possible to characterise the relationship between vasogenic oedema and reduced tracer distribution. Given that ionic and vasogenic oedema increase normal brain tissue volume and, in the event of failed intracranial compliance, an increase in ICP, it may play a role in disrupted CSF and ISF dynamics after stroke as explored in the next section.

### Intracranial pressure and CSF and ISF movement: the chicken or the egg?

**Fig. 2:**
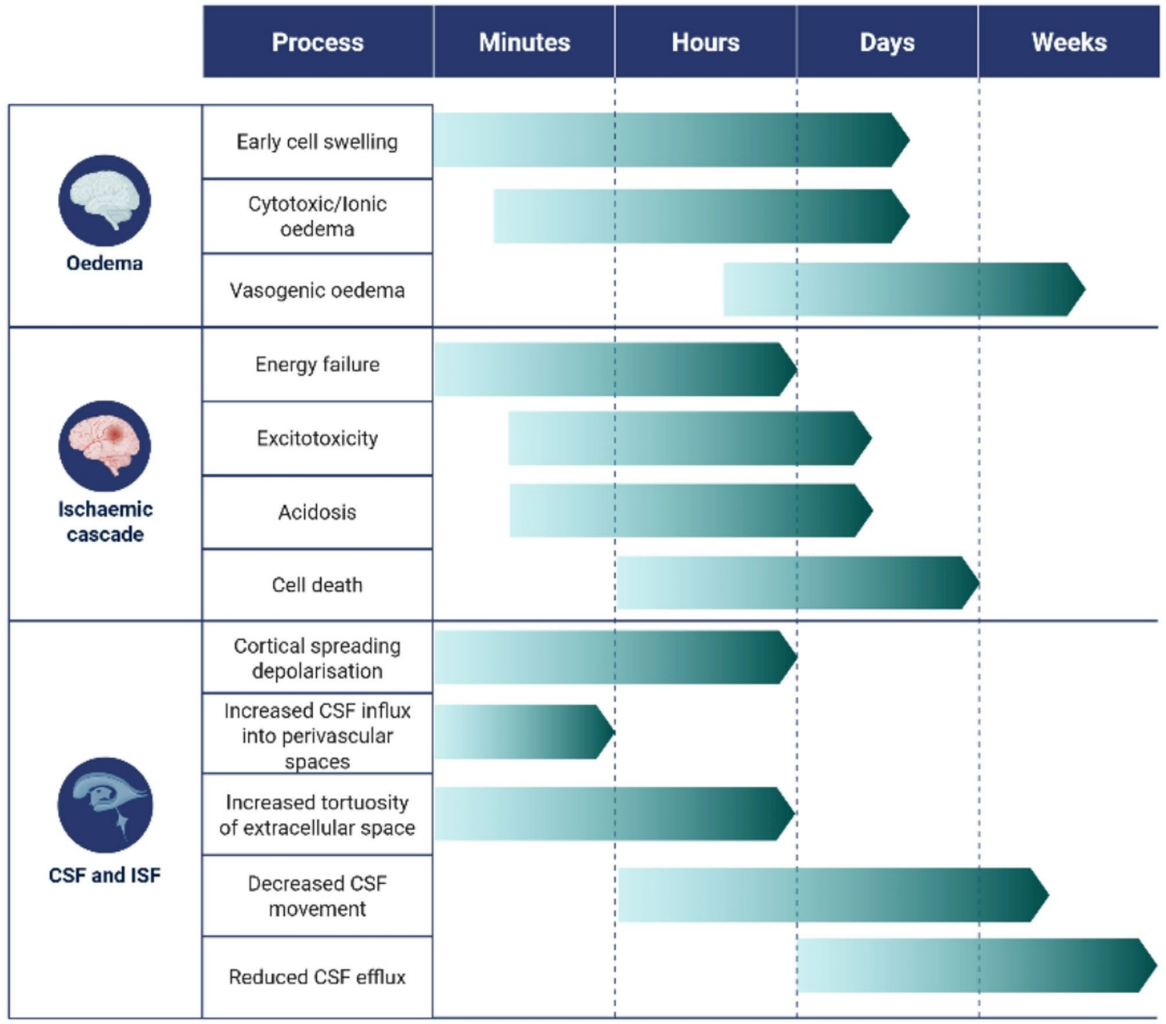
A putative timeline of the events that impact on CSF and ISF movement and exchange in the context of ischaemic stroke. Cortical spreading depolarisation events start within minutes of loss of blood supply and appear to correspond with vasoconstriction and the influx of CSF into the perivascular space [58]. This potentially occurs alongside increased tortuosity of the extracellular space due to cytotoxic cell swelling [150–152] which may slow ISF transit and restrict CSF and ISF exchange in this space. There is an overall decrease in movement of CSF in the perivascular space and penetration of CSF tracers into the parenchyma within hours of a stroke event [32, 55, 59] and there is also evidence of reduced CSF outflow at similar time points [32, 87, 88]. The reduced efflux of CSF from the cranial compartment corresponds temporally with oedema-independent ICP elevation. Created in BioRender [94–96]. Spratt, N. (2025) https://BioRender.com/y16b797

Elevated ICP after stroke has historically been attributed to oedema, however, oedema is not the only cause of ICP elevation (Fig. 2). Our understanding of ICP and oedema after stroke comes mostly from patients with malignant infarction (a large ischaemic stroke with acute brain swelling within 48 h of injury), a condition characterised by rapid neurological deterioration due to sudden, extreme ICP elevation. These patients have elevated ICP, associated with high volumes of vasogenic oedema, typically peaking 3–5 days post-stroke and which, if untreated, can culminate in a sudden and extreme pre-terminal increase in ICP [94, 95], reviewed by [96]. This sudden pre-terminal increase in ICP is not necessarily due to oedema, with available evidence suggesting that, in many patients, brain herniation due to mass effect preceded the sudden, severe ICP rise in which additional volume of blood and/or CSF surpasses the capacity for cranial compliance. Published investigations of patients with elevated ICP due to malignant oedema demonstrate a reduction in CSF-occupying space due to mass effect, but this does not provide much insight into whether movement of CSF or ISF is reduced [97–99].

Oedema, however, is not the only driver of ICP elevation after stroke when cranial compliance is exhausted or fails. Several studies have reported ICP elevation at ~ 24 h after experimental MCAo that does not correlate with oedema volume indicating that other mechanisms are involved [88, 89, 100–104]. Work by our team identified an oedema-independent elevation of ICP at 24 h after moderate ischaemic stroke in mice [32], rats [89, 100–104] and in a pilot study in humans [105]. To confirm the validity of this finding, we measured oedema using three different methods (wet/dry weight, MRI, and histology) and confirmed that significantly elevated ICP is present after moderate experimental stroke in a manner that does not correlate with oedema [100]. Oedema-independent ICP changes have the potential to exact a detrimental effect on stroke outcome by reducing perfusion to the hypoperfused penumbra. After stroke, normal cerebral autoregulation is lost in the ischaemic territory since vessels are generally maximally dilated. Therefore, perfusion of the ischaemic territory becomes tightly linked to the cerebral perfusion pressure (CPP) [106]. In this setting, CPP is dependent on ICP (CPP = Mean Arterial Pressure– ICP) meaning that any elevation in ICP will result in lower perfusion of the ischaemic territory, unless accompanied by a matching elevation of blood pressure [107]. This secondary perfusion reduction, within the already vulnerable ischaemic penumbra, may lead to expansion of the infarct core into the penumbra.

As outlined earlier, most of the ‘classical’ conditions involving disruption of CSF movement and production involve elevated ICP (normal pressure hydrocephalus being the exception). Given that evidence indicates that oedema is not the main driver of ICP elevation at 24 h after moderate stroke there must be other mechanisms contributing to this elevation. The Monroe-Kellie doctrine states that there are three key volume-occupying components in the cranial space: blood, brain tissue, and CSF [63, 64]. An increase in any one of these must be compensated by a decrease in a least one of the others to prevent elevated ICP. As such, this oedema independent increase in ICP is most likely driven by increased volume of CSF and ISF or blood. Evidence reported thus far indicates that increased volume of CSF is the more likely contributor [88, 89, 91]. For this to be true, there must be either an increase in CSF production or a decrease in CSF outflow from the craniospinal space [108].

**Fig. 3:**
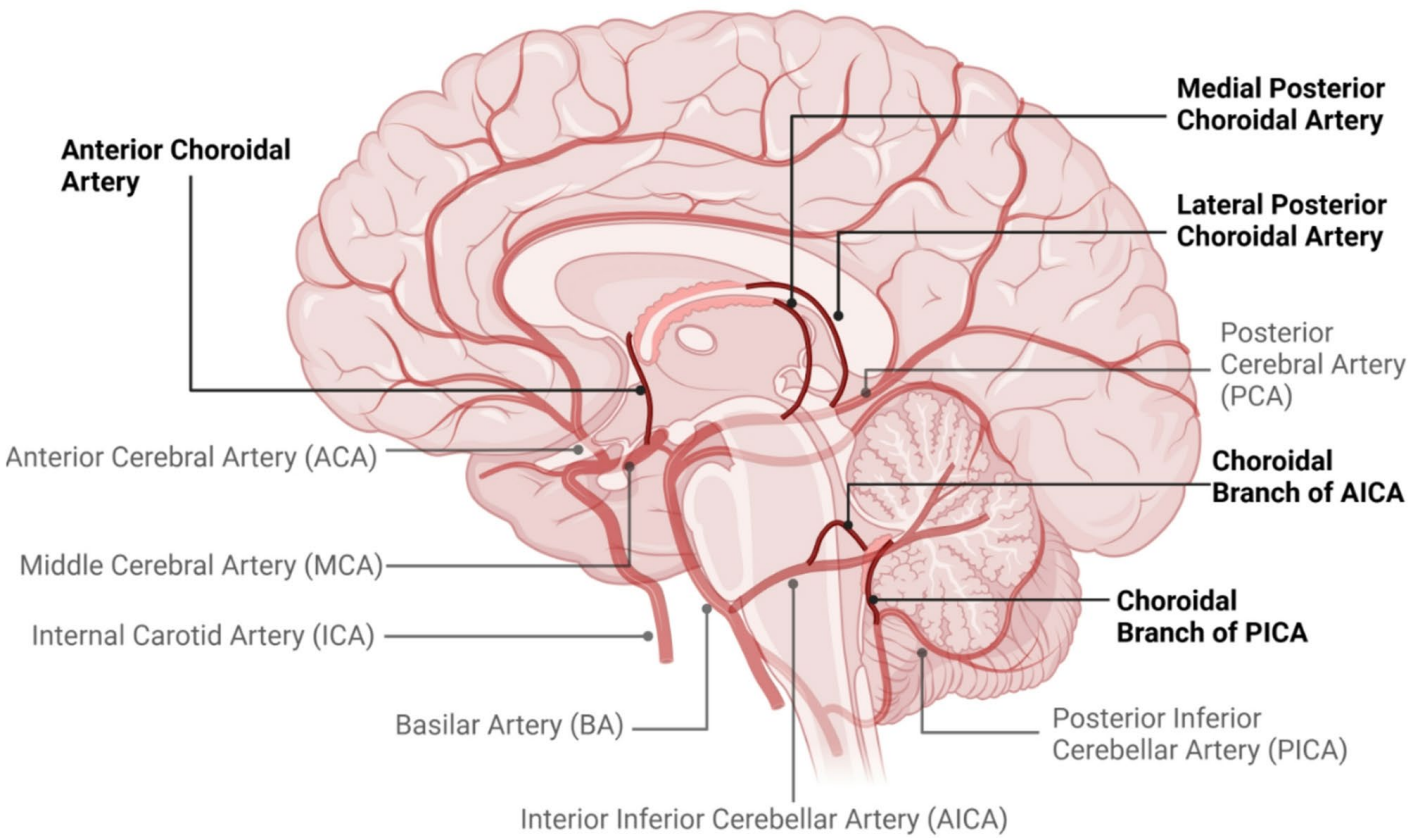
Schematic of the arteries that supply the choroid plexuses of the ventricles. The choroid plexus of the lateral ventricles is supplied by the anterior choroidal artery and posterior choroidal arteries. The posterior choroidal artery also supplies the choroid plexus of the third ventricle. The fourth ventricle choroid plexus is supplied by inferior cerebellar artery. Created in BioRender. Spratt, N. (2025) https://BioRender.com/j95t466

Since CSF is primarily produced by the choroid plexuses, which are primarily fed from branches arising from the MCA, the most common site of ischaemic stroke, damage to the plexuses due to ischaemia may increase CSF production and thus increase ICP. The choroid plexus of the lateral ventricles is supplied by the anterior choroidal artery and posterior choroidal arteries (Fig. 3). The posterior choroidal artery also supplies the choroid plexus of the third ventricle. The fourth ventricle choroid plexus is supplied by inferior cerebellar artery [109]. Na^+^/K^+^-ATPase is one of the key ion transport channel proteins in the choroid plexus [3, 110]. Because its function is dependent on ATP, a hypoxic local environment should lead to reduced CSF production by this channel, but direct evidence of an important effect is lacking. Strokes that affect blood flow to the choroid plexus are relatively uncommon and their effect on CSF production is unclear. Previous work by our team in experimental stroke, determined there was no significant difference in damage to the choroid plexus between groups with or without a patent anterior choroidal artery at 72 h after experimental MCAo, indicating that an alteration to CSF production at this time point is unlikely [111]. Observations made by other teams at earlier timepoints reported that reduced perfusion of the choroid plexus was associated with oedema of this tissue in Sprague Dawley rats 24 h after MCAo, ostensibly due to observed disruption of the blood/CSF barrier of the choroid plexuses of the lateral ventricles in more severe models of choroid plexus hypoperfusion [112, 113]. Increased leakiness of the blood/CSF barrier could indicate increased transit of fluids and solutes into the CSF after stroke, which would, theoretically, increase the volume of CSF or alter the osmolality. Damage to the choroid plexus after stroke appears to be transient, with damage being repaired between 12 and 72 h after ischemia [114, 115], indicating that any alterations to choroid plexus function, and therefore CSF production, are relatively short lived. One study that examined CSF production after stroke reported that it was reduced in mice 200 min after stroke [59]. Meanwhile, another study reported no difference in CSF production 24 h after stroke in rats [88]. Whether these fluctuations in CSF production correspond to choroid plexus damage and repair is yet to be examined. One interesting study examined changes in choroid plexus and lateral ventricle volume in a cohort of stroke patients. Using MRI, the investigators found that all stroke patients had enlarged ipsi- and contralateral choroid plexuses compared to non-stroke controls six weeks, and three and 12 months after stroke [116]. They also noted that the lateral ventricles increased in volume with time post stroke. While ventricular enlargement after stroke is not unusual, with hydrocephalus ex vacuo (loss of brain tissue resulting in enlargement of the ventricles) occurring due to brain tissue atrophy, the chronic enlargement of the choroid plexus is unexplained. Given that the choroid plexus remained enlarged in stroke patients, it is possible that this enlargement is epiphenomenal or occurred prior to the stroke, particularly given the increased incidence of hypertension in the stroke cohort. Given that this study was unable to examine CSF production after stroke, it is not possible to draw clear conclusions about any potential alteration of CSF production.

Additional work by our team and others indicates that increased outflow resistance, and thus lower CSF clearance, may contribute to post stroke ICP elevation. While the relationship between reduced CSF clearance and ICP elevation have long been reported in other conditions [108], the absence of a correlation between ICP and oedema in these stroke studies [100], combined with a lack of evidence of increased CSF production [59, 88] indicate that there is likely a reduction in CSF outflow after stroke. Alshuhri et al., [88] used a modified steady-state infusion method whereby artificial CSF was infused into the lateral ventricles of MCAo or sham Wistar Kyoto rats ~ 25 h after surgery while ICP was monitored [88, 108, 117]. In stroke animals there was increased outflow resistance relative to sham operated animals. Work by our own team comparing animals exposed to hypothermia, which has previously been demonstrated to prevent ICP elevation [103, 118], to normothermic MCAo animals, showed a non-significant trend to lower CSF outflow resistance in the hypothermia treated animals, with the point estimate for outflow resistance more than 2/3 lower than in the normothermic animals [89]. In separate studies, our team identified reduced CSF tracer clearance to the deep cervical lymph nodes [91] and cribriform plate [32], two of the key sites of outflow from the cranial space, at 18 and 24 h after ischaemic stroke respectively.

Studies examining CSF outflow in the context of stroke have rarely examined the role of spinal sites of outflow despite evidence that this is a site where CSF outflow occurs under normal conditions [25, 90, 119–121] (reviewed by [18]). This area is of particular importance in the context of elevated ICP after stroke given earlier reports indicating that outflow of CSF at spinal sites only occurs when CSF tracer is subject to elevated pressure [122–125]. Bothwell et al., [91] reported reduced transit time of CSF tracer to the spinal canal after photothrombotic stroke in rats, which may indicate that CSF preferentially exits via spinal nerve roots and arachnoid granulations rather than exiting via cranial routes after stroke. Increased outflow at spinal exit sites has been reported in studies where ICP was elevated in a mouse model of brain tumours [126]. In that study, fluorescent tracer injected into the CSF was found to preferentially exit at the sacral spine and there was minimal outflow from the cranial exit routes. Another study reported similar findings when ICP was elevated by inflating an epidural Fogarty catheter balloon. In this study, elevated ICP led to reduced CSF outflow to the deep cervical lymph nodes and increased outflow to the sacral lymph nodes, indicating that alternate routes of outflow may become dominant when ICP is elevated [127]. Increased spinal outflow could ostensibly lead to a change in pressure gradient which might explain why Bothwell et al., [91] saw reduced transit time to the spinal column after stroke in the presence of elevated ICP. It should be acknowledged that the circulation of CSF within the spinal column is complex and significantly influenced by respiration, likely acting through venous volume effects [128]. Thus, experimental studies in anaesthetised animals, even if freely breathing, as in the above-mentioned studies, may not completely represent the situation in stroke patients. Whether potential spinal exit routes exhibit increased outflow to compensate for the decreased outflow and raised ICP noted in the cranial space is yet to be examined, however, any compensatory outflow seems to be insufficient to prevent ICP elevation [88, 89]. This makes sense in the context of work by Marmarou et al., [120] who reported that the spinal axis accounts for only 32% of total CNS compliance.

Cumulatively, the evidence indicates that increased resistance to CSF outflow, resulting in accumulation of CSF and ISF in the cranial compartment, is potentially one mechanism contributing to elevated ICP after stroke. This is in line with the work of Davson and Segal [129], and Marmarou et al., [120] who stated that, assuming a constant rate of CSF production, increased CSF outflow resistance is an important contributor to ICP elevation. Elevated ICP has been demonstrated to decrease perfusion of the ischaemic territory in stroke [111] and it is feasible that this leads to expansion of the stroke core. Decreased upstream mixing of CSF and ISF could further exacerbate secondary damage by reducing efficient clearance of osmolytes released as a result of stroke injury. Thus, CSF efflux from the CNS is likely an important contributor to stroke expansion and potentially secondary damage, which are novel targets that could inform the development of new therapeutics.

### Evidence that cortical spreading depolarisation disrupts CSF and ISF movement

Cortical spreading depolarisations (CSDs) are waves of depolarisation that propagate across the cortex [130]. This depolarisation results in release of intracellular K^+^ and neurotransmitters, and concomitant uptake of Na^+^ and Cl^−^. In healthy tissue, neurons are repolarised by ATP-dependent expulsion of Na^+^ and Ca^2+^, but in ischaemic conditions this ATP-dependent transport fails. CSDs are thought to be the process responsible for migraine aura, and may occur in response to many different forms of naturally-occurring or experimentally induced cortical injury [131]. CSDs that occur in hypoperfused tissue result in iso-electric spreading depressions referred to as peri-infarct depolarisations (PIDs) [132]. Waves of PIDs in the hypoperfused tissue of the penumbra lead to increased energy demands and can result in persistent depolarisation in the absence of sufficient ATP to drive Na^+^/K^+^ ATPase function. If perfusion to the penumbra is not restored in a timely manner, cells experience extended periods of depolarisation, resulting in cell death [133]. These waves of depolarisation can also spread into adequately perfused tissue, where the resulting depolarisation events are transient, but can feed back into the hypoperfused tissue, further exacerbating supply mismatch. The number of spreading depolarisation events correlate with infarct size, indicating that a greater number of depolarisation events worsen stroke outcome [134–140]. There may be, however, other mechanisms by which CSDs and PIDs can impact on stroke outcome, particularly in the context of CSF and ISF movement and exchange.

Depolarisation in the infarct core occurs due to energy failure. Neuronal cells take up CSF and/or ISF alongside Na^+^ and Cl^−^, and release K^+^ and glutamate into the ECS [141]. This results in two things: (1) Swelling of cells as a result of CSF and/or ISF uptake, and (2) Reduced ECS and concentration of its contents due to cell swelling [138, 142, 143]. The elevated K^+^ and glutamate in the ECS precipitates depolarisation and swelling of nearby cells and thereby initiating the formation of cerebral oedema.

An additional, and particularly damaging effect in the context of stroke, is the constriction of blood vessels as a result of cellular depolarisation which further exacerbates supply mismatch [144–146]. As recently reviewed by Hladky and Barrand [66], Mestre et al., [59] demonstrated a rapid influx of CSF fluorescent tracer into the periarterial space in the minutes following stroke that appeared to be driven, in part, by CSD-derived vasoconstriction. This vasoconstriction resulted in dilation of the perivascular space, allowing CSF tracer to flood into it. Given that other studies note a reduction in CSF and/or ISF tracer influx at later time points it is clear that this influx of tracer does not persist outside the hyper-acute phase. It also raises the question of what happens to the CSF tracer after its early, rapid influx into the perivascular space. One possibility is that the tracer in the perivascular space moves into the ECS where it mixes with ISF, but this was not examined by Mestre et al., [59] meaning it is not possible to draw a firm conclusion. Another possibility is that the vasodilation that follows vasoconstriction, seen from 15 to 30 min after the CSD wavefront, could prevent the entry of CSF tracer injected at later time points (i.e., 3 h) into the perivascular space. Whether CSDs that occur beyond 30 min after stroke result in the same influx of CSF into the perivascular space has not been examined. A study examining the perivascular space during and after CSDs verified that the perivascular space dilates in concert with depolarisation-derived vasoconstriction and that subsequent vasodilation shrinks the perivascular space [147]. This study also demonstrated that a fluorescent CSF tracer does not appear to enter the parenchyma after shrinkage of the perivascular space, indicating that tracer in the perivascular space at the time of vasodilation, and concomitant shrinkage of the perivascular space, could potentially be pushed back out into the subarachnoid space [147]. If there are repeated CSDs, as is known to occur days to weeks after ischaemic stroke [134], there may be multiple timepoints when dilation and constriction of cerebral arteries and arterioles results in the inverse change in volume of the perivascular space. This could explain the reduced perivascular load of tracer noted by Wang et al., [57] and Arbel-Ornath et al., [58] in the days following ischaemic stroke.

### Impacts of ischaemic stroke on ISF movement and exchange with CSF in the parenchyma

Another potential reason for the decrease in CSF and/or ISF tracer movement noted in the above papers is altered microstructure of the ECS in the central nervous system. The ECS is made up of 20–60 nm wide channels of space between closely packed cells [148] and makes up 20% of normal brain volume [148]. As mentioned earlier, the weight of evidence indicates that diffusive forces dictate ISF tracer movement, although there is ongoing debate in this arena [10]. The rate of diffusion in the ECS is affected by the geometry and composition of this space, with extracellular matrix molecules, binding sites and charge states all affecting the movement of solutes in this space [149]. Regardless of the mechanisms driving the movement of ISF, alterations to the structure of this space would affect how ISF moves. Thorne and Nicholson [150] found that the ECS shrinks immediately after terminal ischemia [150]. Several other studies also reported an increase in ECS tortuosity from ~ 1.6 to ~ 1.9–2.1 in ischaemic conditions [151, 152]. This corresponds to a shrinkage of the ECS from 20% of brain volume to ~ 5–10% [151, 153]. This fits with what is seen in in vivo models of rapid cell swelling due to cytotoxic oedema following ischemia [154–157]. Hrabětová et al., [152] reported that the increase in tortuosity was due to an increase in dead space microdomains. They found that filling these dead space microdomains using large molecular weight solutes reduced tortuosity. In addition, a reduction in the apparent diffusion coefficient of water has been reported to occur within minutes of ischaemia or brain trauma [71, 158–160]. Combined, the above studies indicate that ischaemic conditions, such as those found after ischaemic stroke, increases ECS tortuosity and slows the movement of ISF borne solutes. Evidence indicates that ECS shrinkage is specific to hypoxic tissue, however, studies conducted thus far have examined global models of ischaemia and cannot be used to investigate whether reduced ECS is specific to the ischaemic territory or whether it also occurs outside in non-ischaemic territories within the same brain.

The impacts of ischaemic stroke on the ECS are further confounded by evidence that elevated ICP is partially compensated for by a reduction in cell volumes, which would theoretically result in expansion of the ECS. Kalisvaart et al., [161] reported reduced CA1 and S1 neuron volume, but not striatal neuron volume, in the contralateral hemisphere when compared to sham surgery controls 24 h after MCAo in Sprague Dawley rats. They report that the MCAo model used in this study results in significant oedema, however oedema volumes were not reported and ICP was not measured, ostensibly to maintain the integrity of the cranial compartment. What happens to cell volume in the presence of oedema-independent ICP elevation remains to be determined. Given these findings, it will be important to determine whether the ECS shrinks after ischaemic stroke, and whether any ECS shrinkage is offset by cell volume compliance to determine how ischaemic stroke impacts on ISF movement in the ECS.

It is also a possibility that it is not just the ECS that induces changes to the distribution of ISF tracers in the parenchyma. Tracers entering the parenchyma from the subarachnoid space must cross the pia mater and glia limitans. The pia mater has a low transmembrane resistance making it similar to a ‘leaky’ epithelium [162] as such it does not normally function as a tight barrier. Recent work found that the pia mater of the spine is permeable to macromolecules (a maximum of 66 kDa was examined) but the glia limitans prevents these macromolecules from entering the spinal parenchyma [163]. Smaller 3 kDa solutes were able to cross both the pia mater and the glia limitans to enter the spinal parenchyma. Together, this data indicates that small solutes can cross the pia mater and glia limitans under normal conditions, but, as seen in several studies, this does not happen after stroke. Potentially some property of the pia mater and glia limitans, such as swelling of astrocyte end feet, is affected by ischaemic stroke. Whether such changes that reduce CSF movement across the pia mater and glia limitans occur after ischaemic stroke remains to be investigated but it is clear that ischaemic conditions affect the ECS microstructure in a manner that would reduce transit and exchange of CSF and ISF [151, 152].

### Arterial pulsatility, CSF and ISF dynamics, and ischaemic stroke

Acute and chronic disturbance to arterial pulsatility and blood pressure that occur prior to, during, and after stroke could ostensibly result in disruption to CSF movement. Instantaneous CSF movement is predominantly driven by cardiac and respiratory cycles [19, 31, 164] and may be reduced in hypertension– a major risk factor for stroke. The few studies that have examined CSF movement in the presence of hypertension have reported mixed results. One study reported an ~ 40% decrease in mean periarterial CSF flow velocity during pharmacologically induced hypertension in mice [31]. These results can appear somewhat counterintuitive given that hypertension is associated with increased arterial pulsatility, which should, ostensibly, result in increased CSF velocity in the periarterial space. Mestre et al., [31] reported that this is likely due to hypertension generating increased backflow, where the CSF moves from downstream to upstream, compared to normotensive conditions. In their study, backflow was determined to be present when microspheres in the perivascular space of pial vessels had a negative velocity, indicating that they were moving in the opposite direction to the measured direction of flow. Whether this indicates a change in net flow of CSF is unclear. It is also important to note that the findings reported by Iliff et al., [19] and Mestre et al., [31] were recorded in subarachnoid/pial vessels and may not reflect the patterns seen in the perivascular space of penetrating arterioles. These findings are particularly interesting given that another study found that ISF drainage from the parenchyma was not altered in the presence of phenylephrine-induced hypertension in mice [58] indicating that while there may be a reduction in influx of CSF in the perivascular space, the mechanics of ISF movement from the parenchyma appear to remain unaffected by hypertension. It remains to be seen, however, whether the differences observed in these two studies is due to differences in the methods and models used. In addition, it remains to be examined whether changes to CSF and ISF movement in pharmacologically induced hypertension are reflective of those in models and patients with chronic hypertension given that chronic hypertension is associated with increased arterial stiffness [165] and arterial pulse wave velocity [166, 167].

Arterial pulsatility is decreased in occluded vessels and should, theoretically, coincide with reduced CSF movement in the periarterial space [168]. This fits with the findings of Gaberel et al., [56] and Arbel-Ornath et al., [58] whose work highlighted the possibility that vessel patency is important in normal CSF and ISF tracer distribution [56, 58]. Gaberel et al., [56] found that after spontaneous recanalization (confirmed using magnetic resonance angiogram) of the MCA, CSF tracer distribution was comparable to sham operated animals at 24 h after occlusion. Arbel-Ornath et al., [58] reported similar findings, with migration of ISF tracer from the ECS to the periarterial space being reduced in occluded vessels compared to non-occluded vessels within the ipsilateral hemisphere. Other studies, however, have reported a return to baseline CSF tracer distribution while vessels remain occluded. Warren et al., [32] reported that CSF tracer distribution was comparable to that seen in sham-operated animals at 14 days post stroke in a photothrombotic model of ischaemic stroke, even though vessels remain occluded. Wang et al., [57] noted a similar temporal relationship, however, given that theirs was a multiple microinfarct model induced using cholesterol crystals, it is possible that by day 14 the cholesterol crystals had dissolved, restoring vessel patency. It is important to note when considering the results reported by Wang et al., [57] that the multiple microemboli stroke model used in their study could result in occlusion of multiple arterioles, of which, not all will result in infarction. The relevance of this model to thrombo-embolic ischaemic stroke is debatable as it is more similar to an embolic shower and so, its findings on the impact of CSF and ISF movement are not clear cut.

Together this suggests that restoration of normal CSF and ISF tracer movement may not be dependent on recanalization of the occluded vasculature alone, but it is clear that when examining CSF and ISF after stroke, it is important to consider the impacts that cerebrovascular physiology has on the system. Our incomplete understanding of how these two systems interact, and how variations such as hypertension can impact on this interaction, makes it challenging to understand implications for ischaemic stroke severity and outcomes.

### Influence of stroke severity on CSF movement

There is little evidence to suggest that the severity of stroke injury is an important determinant of the duration of disrupted CSF or ISF movement and exchange. The restoration of normal CSF and ISF tracer distribution between three and 24 h after occlusion as seen by Gaberel et al., [56], is earlier than seen in other studies, which reported ongoing disruption to tracer distribution at 24 h and three days respectively [32, 57]. While Gaberel at al., [56] do not report lesion volume in their study, their study design is such that the MCA may have spontaneously recanalized any time between 60 min (occlusion confirmation) and 24 h (follow-up imaging) [56]. As such, it is feasible to speculate that the MCA was occluded for only a short duration for at least some animals, resulting in smaller lesion volume. Warren et al., [32] reported a global disruption to CSF tracer distribution 24 h after unilateral photothrombotic cortical stroke. The photothrombotic model used in the study resulted in a focal lesion of small to moderate volume (7.6 ± 2.14 mm^3^ at 24 h after stroke, 3.35 ± 1.32 mm^3^ 14 days after stroke), which, if lesion volume was an indicator of the extent of reduced CSF movement, would be an unexpected outcome [32]. Furthermore, the reduction in CSF tracer infiltration of the parenchyma persisted until at least 24 h after occlusion, indicating that even small to moderate volume lesions can decrease CSF movement beyond the acute phase of stroke. In further support of the lack of relationship between stroke severity and extent of disruption to CSF movement, one study examining stroke patients with futile recanalization found there was no association between infarct volume and CSF transit in the perivascular space [62]. Further work to understand whether lesion volume, and thus stroke severity, correspond to a longer duration of disruption to CSF and ISF movement is warranted.

### Aquaporin 4 (AQP4)

Another putative molecule important in maintaining CSF and ISF exchange is the water channel aquaporin 4 (AQP4) and there is evidence to indicate that, while the abundance of AQP4 does not appear to be altered after stroke, the location of AQP4 in the lipid membrane of astrocytes is changed. AQP4 allows for bidirectional movement of water and its expression in the central nervous system is restricted to astrocytes of the brain and spinal cord, and the ependymal cells of the cerebral ventricles [169]. In particular, AQP4 is found concentrated on astrocyte end feet which encapsulate the perivascular space [5]. In their original studies, Iliff et al., [14], hypothesised that AQP4 transports water into the parenchyma in a trans-astrocytic manner and in doing so establishes the osmotic gradient that permits convective transport of CSF solutes into the parenchyma.

As such, studies that examine AQP4 in ischaemic stroke models tend to focus on the role of this water channel in oedema. AQP4 knock-out mice and wildtype mice treated with AQP4 inhibitors have been demonstrated to be protected from oedema after stroke [170–173]. Yao et al., [170] report that AQP4^−/−^ mice had reduced oedema and lesion volume when compared to AQP4^+/+^ mice 24 h after stroke. Furthermore, they demonstrate that in the absence of AQP4, there is reduced extravasation of the large molecular weight dye, Evans Blue, from the vascular compartment. Under normal circumstances Evans Blue does not cross the BBB. This indicates that AQP4 could play a role in the development of vasogenic oedema. Other studies have reported altered distribution of AQP4 after stroke such that it is no longer localised to perivascular end feet [60, 174, 175], potentially a response to the cellular influx of water that occurs during cytotoxic oedema. Using models with a similar alteration in AQP4 distribution, other investigators have also reported reduced parenchymal infiltration of CSF tracer in healthy animals [176–178]. The altered localisation of AQP4 after stroke could explain some of the reduction in CSF tracer distribution. It is possible that re-localisation of AQP4 to astrocyte end feet may coincide with restoration of CSF tracer distribution to pre-stroke levels, but this is yet to be investigated. In addition, swelling of astrocytic end feet during cytotoxic oedema may reduce the size of the gaps between the end feet, restricting movement of solutes from the perivascular space to the interstitial space [129, 179–181]. This may be an explanation for the reduced parenchymal CSF tracer load after stroke reported in several studies [32, 56, 57].

## Potential impacts of altered CSF and ISF movement and exchange on stroke outcome and future directions

**Fig. 4:**
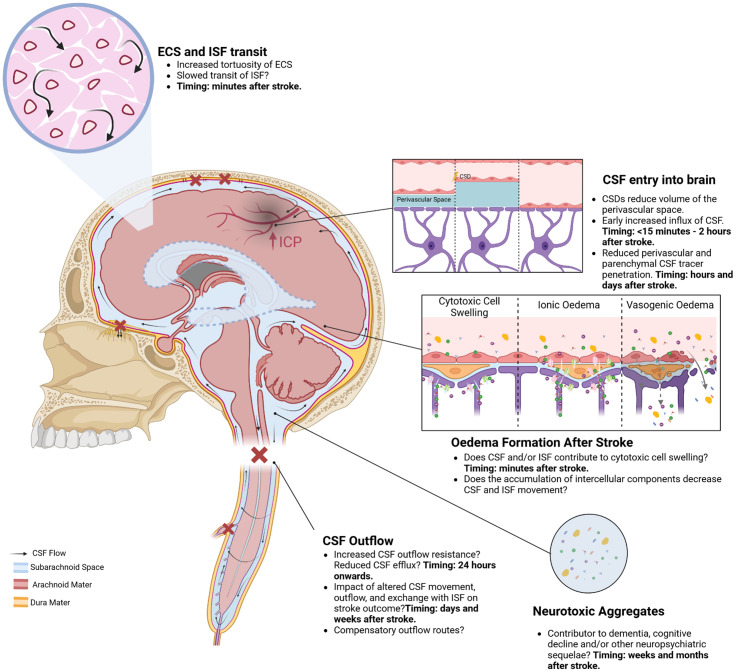
A schematic summarising the ways that ischaemic stroke impacts on CSF and ISF movement and efflux from the cranial compartment. Ischaemic stroke triggers cortical spreading depolarisations (CSDs) which are associated with an initial increase, and then decrease, in the perivascular space that appears to initially increase, and then restrict, CSF movement into this space. There is also evidence that there is increased tortuosity of the extracellular space (ECS) which hinders the movement of ISF-bourne solutes. This overall reduction in the movement of CSF and ISF might result in reduced clearance of waste products which may contribute to the increased prevalence of cognitive decline noted in stroke patients. CSF and ISF are likely contributors to early cell swelling and cytotoxic/ionic oedema, which may exacerbate vasogenic oedema. Furthermore, there is evidence that CSF outflow is reduced at the cribriform plate and potentially other sites which may lead to elevated intracranial pressure (ICP) and reduced perfusion of the at-risk penumbra. Created in BioRender. Spratt, N. (2025) https://BioRender.com/y16b797

The studies examined in this review indicate that there is a decrease in CSF exchange with ISF, and a decrease in the circulation, and outflow of these fluids in the hours and days after stroke. Disrupted CSF and ISF movement and exchange may contribute to the severity of stroke by contributing to infarct expansion and reducing system capacity to restore CNS homeostasis (Fig. 4).

In the context of oedema; the work of Mestre et al., [59] demonstrated an influx of CSF in the minutes after a stroke that may contribute to early cell swelling and the formation of cytotoxic oedema (although it should be noted that there is debate as to whether oedema that occurs in the minutes following stroke constitutes cytotoxic oedema) [59, 66]. This influx, combined with the release of intracellular components into the interstitial space due to energy failure and subsequent cell death, leads to cell swelling. This accumulation of intracellular components in the interstitial space is likely exacerbated by the overall decrease in movement of CSF and ISF noted from three hours after stroke [32, 56, 58, 60]. The bulk movement of water and osmolytes from the intercellular space to the intracellular compartment drives formation of an osmotic gradient that contributes to the development of cytotoxic/ionic oedema [65]. The size of the osmotic gradients that arise during stroke are only partially characterised in experimental models of stroke. Cytotoxic/ionic oedema, and in turn vasogenic oedema, represent a net gain of volume within the cranial compartment which, if it exceeds the capacity for compliance, leads to elevated ICP. ICP reduces cerebral perfusion pressure and blood flow to the stroke site, worsening ischemia within the territory of the occluded vessel and potentially contributing to delayed infarct expansion [111]. Thus, altered movement of CSF and/or ISF and its potential role in the formation of cytotoxic oedema after ischaemic stroke may contribute to the formation of ionic and vasogenic oedema which in turn is associated with worse stroke outcomes.

CSF outflow also likely plays a pivotal role in oedema-independent ICP elevation. Evidence indicates that oedema-independent ICP elevation is driven by increased CSF outflow resistance [88, 89]. As outlined previously, arteries within the ischaemic brain tissue are maximally dilated and thus perfusion is dictated by cerebral perfusion pressure. As cerebral perfusion pressure is a function of the mean arterial pressure minus the ICP, elevated ICP reduces blood flow to hypoperfused tissue, potentially contributing to stroke core expansion into the otherwise salvageable penumbra.

Evidence of reduced CSF and ISF circulation and outflow after ischaemic stroke highlights the potential that reduced CSF exchange with ISF, which is posited to be an important ‘rinsing’ mechanism for the brain, may result in debris and waste from stroke injury remaining in the cranial space longer than would otherwise be the case. This could exacerbate acidosis and excitotoxicity and potentially contribute to cognitive decline and/or other neuropsychiatric sequelae such as depression and fatigue. Stroke patients are 1.8 times more likely to develop dementia than non-stroke individuals [182]. Why stroke patients are more at risk for dementia is likely multifactorial, but there is the potential that stroke-mediated disruption to CSF and ISF movement and exchange, and reduced CSF efflux from the cranial compartment after stroke could be an important contributing factor. Numerous pre-clinical studies have reported accumulation of amyloid β [14, 33, 177, 183] and other neurotoxic protein aggregates [178] in the presence of reduced movement of CSF and/or ISF. Furthermore, amyloid β accumulation has been reported in ~ 24% of stroke patients with cognitive impairment in a small cohort, but it is not clear whether this amyloid β deposition was present prior to the stroke event [184]. The delay in clearance of intrathecal tracer in a cohort of dementia patients when compared to matched controls reported by Ringstad et al., [36], indicates that CSF outflow is reduced in these patients and the authors posit that this may be part of the reason for the accumulation of neurotoxic aggregates. Given that we see both a decrease in efflux and circulation/exchange of CSF and ISF in most experimental stroke models, it is within the realms of possibility that these disruptions contribute to the increased risk of neurodegenerative disease in stroke patients. The relationship between reduced CSF and ISF exchange and outflow from the cranial compartment will need to be carefully teased apart due to the presence of several shared co-morbidities between stroke and neurodegenerative disease to establish a connection.

There is evidence that CSDs alter the volume of the perivascular space, by first expanding and then shrinking, in a manner that alters CSF distribution. At present, the interaction between CSDs and CSF dynamics have only been examined in the 30 min immediately following stroke. Examination of additional timepoints is needed to further expand our understanding of the role of CSDs in disrupting CSF, and potentially ISF movement, after stroke. Similarly, changes to the microstructure of the parenchyma have been examined in global ischemia, and it has been demonstrated to lead to increased tortuosity that slows transit of ISF tracers. How ischaemic stroke impacts on the architecture of the intercellular space has not been examined in any detail and it would likely provide insight into the reduced distribution of ISF tracer in the parenchyma noted after stroke.

In addition, our incomplete understanding of the routes of CSF efflux from the cranial compartment, whether outflow is reduced at all sites of efflux after stroke, and what the mechanisms are that underly this decrease in outflow need to be determined to allow for the development of effective therapeutics. This, as well as a better understanding of when baseline CSF and ISF movement is re-established after stroke will provide significant insights into how CSF and ISF exchange, movement and CSF outflow impacts on stroke outcome. Improvements to in vivo imaging of CSF movement and exchange are imperative to allow for real-time assessment of changes in this system without compromising the cranial compartment. This will be particularly powerful in patient studies.

In conclusion, given the evidence that CSF and ISF exchange and movement is reduced after stroke, even if only temporarily, there is the potential that this reduction in CSF and ISF movement, exchange and CSF outflow from the cranial compartment exacerbates negative outcomes as a result of stroke. If this is the case, CSF and ISF movement represent a hitherto untapped therapeutic target that could be leveraged to improve outcomes for stroke patients. To further understand the impact of altered CSF and ISF movement, outflow, and exchange on stroke sequelae, fundamental studies are needed, examining how stroke severity, location, and stroke model alters CSF and ISF dynamics.

## Data Availability

No datasets were generated or analysed during the current study.

## Abbreviations

CPP: Cerebral Perfusion Pressure
ECS: Extracellular Space
ICP: Intracranial Pressure
IPAD: Intramural Periarterial Drainage
ISF: Interstitial Fluid
MCA: Middle Cerebral Artery
MCAo: Middle Cerebral Artery occlusion
PI: Pulsatility Index
